# Soft Tissue Surgical Technique for Obligate Dislocation of the Patella

**DOI:** 10.31486/toj.25.0061

**Published:** 2026

**Authors:** Barbara Minkowitz, Nitish R. Kasarla, Colleen M. Spingarn, Julia A. Matalon, Nicholas J. Avallone, Michael T. Busch

**Affiliations:** ^1^ Department of Pediatric Orthopedics, Morristown Medical Center, Atlantic Health System, Morristown, NJ; ^2^ Cooper Medical School of Rowan University, Camden, NJ; ^3^ Department of Orthopedics, Morristown Medical Center, Atlantic Health System, Morristown, NY; ^4^ Department of Pediatric Orthopedics, Children's Healthcare of Atlanta, Atlanta, GA

**Keywords:** *Congenital dislocation of the patella*, *joint dislocations*, *knee joint*, *patellar dislocation*, *postoperative care*, *range of motion–articular*, *surgery*

## Abstract

**Background:**

Obligate dislocation of the patella (ODP) is a subtype of congenital disorders and dislocations of the patella. Severity of the condition varies; symptoms range from minor disabilities to impaired ambulation. Definitive treatment for ODP is surgery, with the choice of surgical procedure determined by skeletal maturity.

**Case Series:**

This report documents a soft tissue surgical correction for 2 patients with ODP using (1) lateral release of the retinaculum, (2) Z-lengthening of the quadriceps tendon, (3) patellar tendon split transfer (medial side only), and (4) vastus medialis oblique muscle advancement. Patient 1 is a 7-year-old skeletally immature male who experienced complete lateral dislocation of the right patella with flexion. The patient was unable to play sports without falling and without constant patellar dislocation. Patient 2 is a 7-year-old skeletally immature male with Down syndrome who presented with a right laterally dislocating patella with flexion and a 15° flexion contracture of the knee. The patient was unable to ambulate and—according to his parent—would “hit the side of his patella” to center it on his knee to provide stability and permit weight-bearing in a standing frame. After surgery and recovery, both patients reached full weight-bearing without dislocation. Patient 1 plays sports for the first time and has a range of motion of 165°. Patient 2 can stand with stability, continues to work on gait training, and has a range of motion of 160°.

**Conclusion:**

This combined surgical approach mitigates patellar tilt, knee flexion contracture, and recurrent instability, offering a reproducible option for improving stability, tracking, and long-term function in complex patellofemoral pathology.

## INTRODUCTION

**Figure 1.**
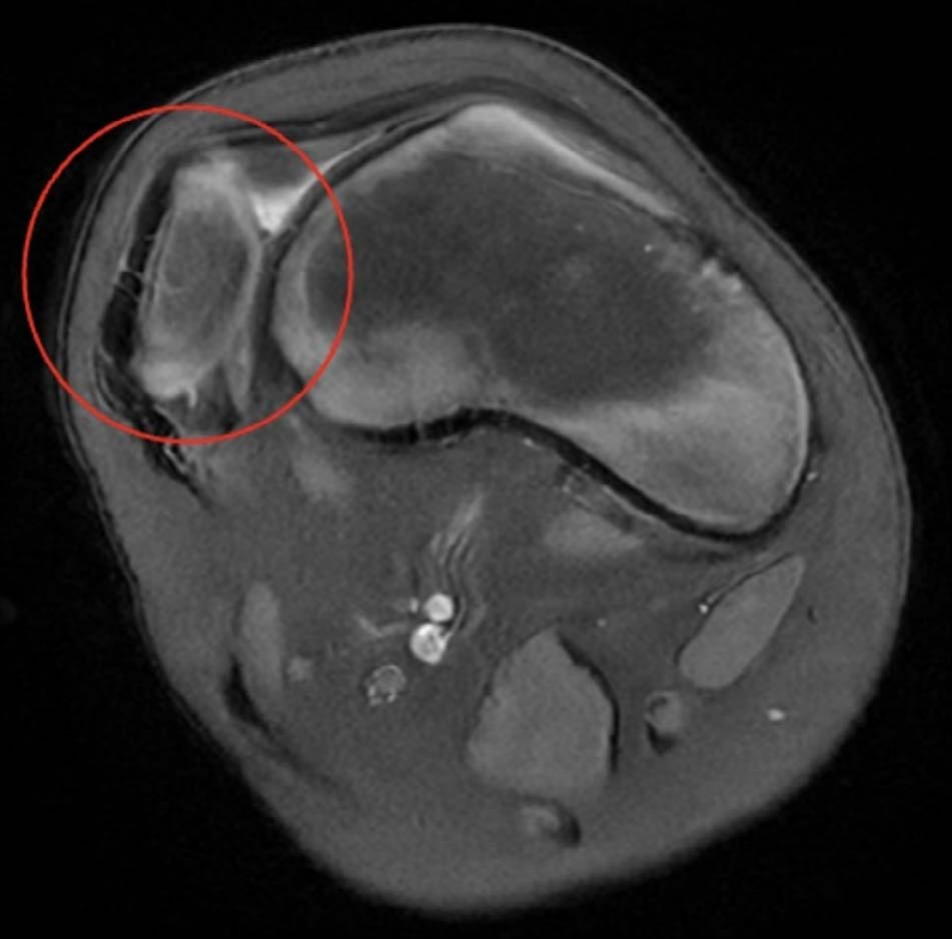
Axial magnetic resonance image shows a dislocated patella (circled in red) with a shallow trochlear groove.

Obligate dislocation of the patella (ODP) is a rare condition in which the patella is intermittently dislocated outside the trochlear groove (Figure 1). ODP is a subtype of congenital disorders and dislocations of the patella. Congenital dislocation of the patella is manually irreducible, whereas ODP is a less extreme condition in which the patella dislocates during flexion and reduces with extension. In some cases of ODP, the patella may stay dislocated in extension, and pressure must be applied to reduce it.^1-8^

Congenital disorders and dislocations of the patella are genetic conditions, with the definitive causes still undetermined. A theorized etiology of patellar dislocations is failure to rotate the quadriceps muscles and patella myotome during gestation—normally occurring during the first trimester—resulting in a misaligned patella and a shallow trochlear groove.^4^ Patellar dislocation conditions can present concurrently with other leg deformities such as femoral retroversion, genu valgum, calcaneovalgus foot deformity, and/or tibial torsion and can be associated with genetic syndromes such as arthrogryposis, Larsen syndrome, dyschondrosteosis, Rubinstein-Taybi syndrome, Down syndrome, and nail-patella syndrome.^6^

**Figure 2.**
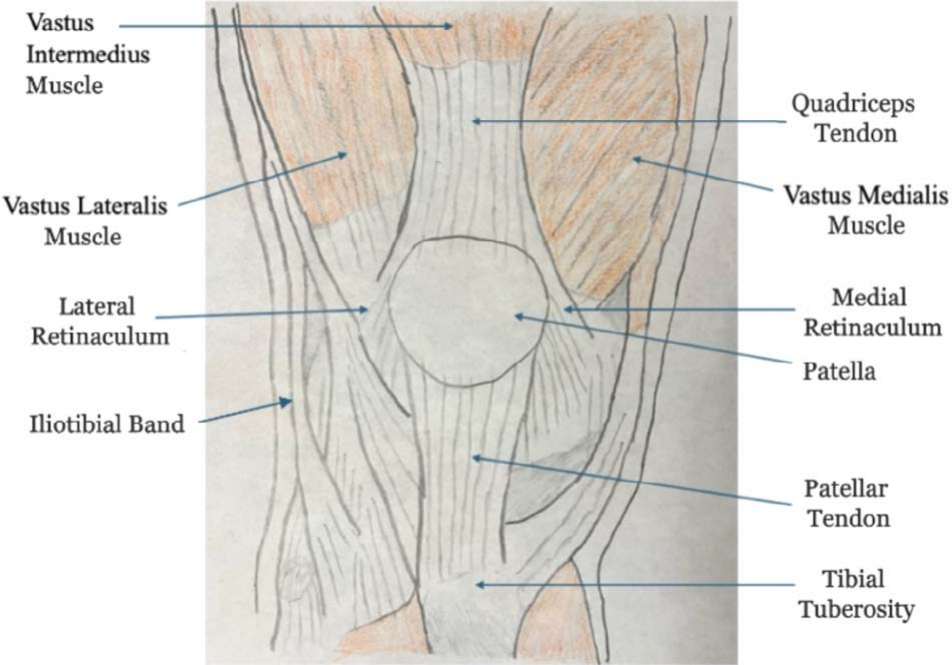
**The diagram depicts the soft tissue structures in the knee.** (Image by Nitish R. Kasarla and used with permission.)

The knee requires several muscles, ligaments, and tendons to produce strong movements. The patella serves as the fulcrum for the quadriceps tendon, with the vastus lateralis, intermedius, and medialis muscles all converging at the quadriceps tendon superior to the patella. In the inferior position, the patellar tendon attaches to the tibia and establishes vital tension, creating the fulcrum of the knee. The lateral and medial retinacula maintain stability with the patella balanced directly over the trochlear groove at the joint. The iliotibial band secures the knee while in extension and flexion (Figure 2).

Abnormalities observed in patients with ODP include iliotibial band inhibition of reduction of the patella; contracture of the lateral capsule and stretching of the medial capsule; loss of knee extension and subsequently, loss of ambulation; and contracture of the vastus lateralis muscle, pulling the quadriceps mechanism to the anterolateral position.^2^

For patients with ODP, casts and braces have been shown to be helpful to improve knee flexion contracture and patellar stability.^5^ However, casts and braces are not a permanent solution. Physical therapy can be helpful but is not curative. Surgery—either soft tissue or bony procedures depending on skeletal maturity—is the only definitive treatment for ODP.^9^

The surgical treatment discussed in this case series is a proximal and distal soft tissue release for skeletally immature patients that includes (1) lateral release of the retinaculum, (2) Z-lengthening of the quadriceps tendon, (3) patellar tendon split transfer (medial side only), and (4) vastus medialis oblique muscle advancement. Previous studies^1-5^ have described less extensive approaches than the approach presented in this case series, and our variations have demonstrated excellent clinical outcomes.

## CASE SERIES

### Patient 1

**Figure 3.**
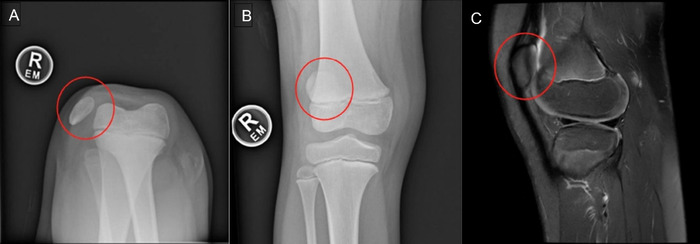
The preoperative knee for Patient 1 is shown in (A) sunrise view, (B) anteroposterior view, and (C) sagittal T2 fat-suppressed magnetic resonance imaging view. In all panels, the patella is circled in red.

Patient 1 is a 7-year-old male with no remarkable medical history. On physical examination, his right patella was completely laterally dislocated but could be fully reduced with extension. No flexion contracture of the knee was present, but the patient presented with minor genu valgum and 40° external tibial torsion. The patient was unable to play sports without falling and without constant patellar dislocation. Radiography and magnetic resonance imaging (MRI) findings confirmed the diagnosis of ODP (Figure 3).

### Patient 2

**Figure 4.**
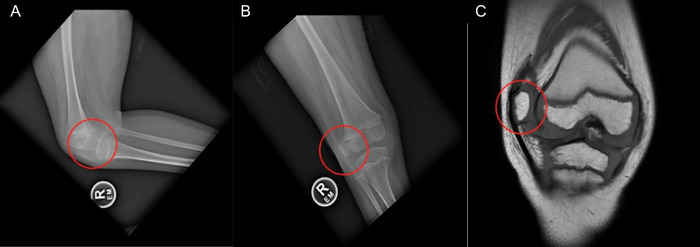
The preoperative knee for Patient 2 is shown in (A) lateral view, (B) anteroposterior view, and (C) coronal T1 magnetic resonance imaging view. In all panels, the patella is circled in red.

Patient 2 is a 7-year-old male with Down syndrome. On physical examination, his right patella was laterally dislocated and could be partially reduced in extension. The patient had a 15° flexion contracture of the knee, minor genu valgum, 30° external tibial torsion, and femoral retroversion. The patient was unable to ambulate, and his parent stated he would “hit the side of his patella” to center it on his knee, thereby providing stability to the leg and permitting weight-bearing in a standing frame. Radiography and MRI findings confirmed the diagnosis of ODP (Figure 4).

### Surgical Treatment

The same soft tissue surgical method was performed on both patients, but the technique is detailed for Patient 2.

**Figure 5.**
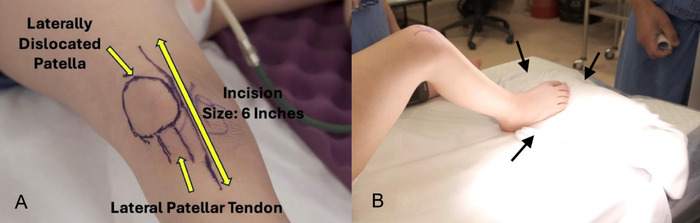
Perioperative planning for the procedure includes (A) marking the knee (arrows indicate the outline of the incision, the laterally dislocated patella, and the laterally positioned patellar tendon), and (B) the placement of a sandbag on the operating table to hold the leg at 60° flexion. Arrows outline the location of the sandbag.

Perioperative planning included marking the knee and taping a sandbag into position on the table so that the leg could be held at 60° flexion when the tendons were reattached for proper tensioning (Figure 5).

**Figure 6.**
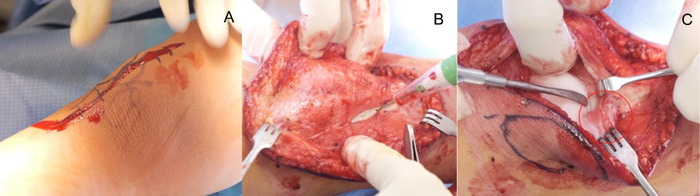
Operative photos show the (A) incision on the knee along the medial aspect of the patella, (B) lateral release of the retinaculum, and (C) visualization of the anterior horn of the lateral meniscus (circled in red). Surrounding structures are retracted to prevent accidental injury to the lateral meniscus.

Using a scalpel and cautery device, an incision was made along the medial aspect of the patella with dissection down to the patella, quadriceps tendon, and patellar tendon (Figure 6A). Use of an Esmarch tourniquet was attempted because of the short segment of the femur, but the tourniquet was removed when it did not fully control bleeding, and a cautery device was used for hemostasis. The retinaculum release was initiated laterally with the cautery device, taking care to visualize, retract overlapping structures, and direct dissection away from the lateral meniscus, protecting it from iatrogenic injury (Figures 6B and 6C).

**Figure 7.**
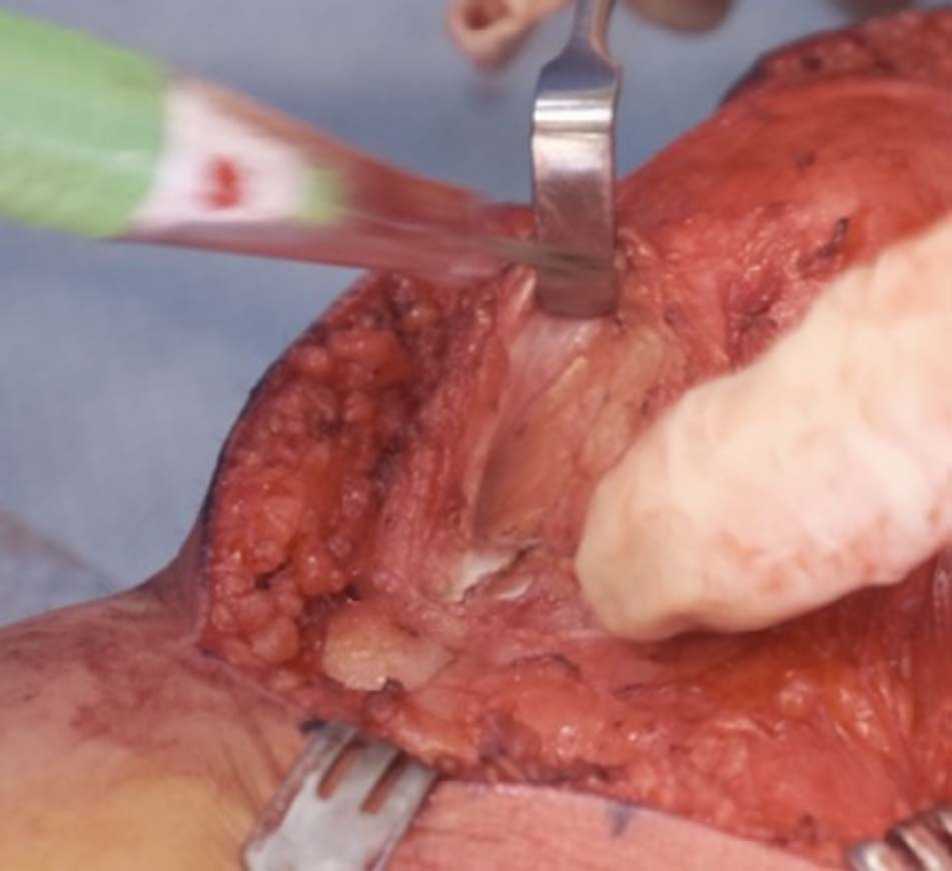
Pulling the patella medially shows the orientation of the knee joint and the soft tissue structures surrounding it.

The lateral retinaculum was released into the quadriceps muscle. The quadriceps muscle was released from the femur and the lateral intermuscular septum using blunt digital dissection, during which minimal tightness was encountered. With the patella pulled medially, the dissection was maintained in the proper anatomic area between the lateral retinaculum and the joint capsule by observing the knee joint orientation in relation to the adjacent soft tissue structures (Figure 7).

**Figure 8.**
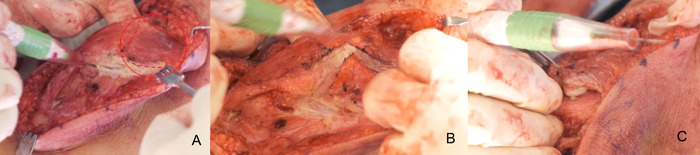
(A) Fibers of the vastus medialis oblique muscle (circled in red) are cut along the red dashed outline to create a sleeve. (B) The vastus medialis oblique muscle cut is detached from the patella. (C) The vastus medialis oblique muscle cut is assessed for sufficient coverage for advancement.

Using the cautery device, the medial patellar tendon was dissected to reveal the fibers of the vastus medialis oblique muscle. The vastus medialis oblique muscle was detached from the patella, leaving a 4-mm sleeve of tissue on the patella to maintain patella vascularity (Figures 8A and 8B). The capsule of the knee joint was maintained below the vastus medialis oblique muscle and assessed for sufficient coverage (Figure 8C).

**Figure 9.**
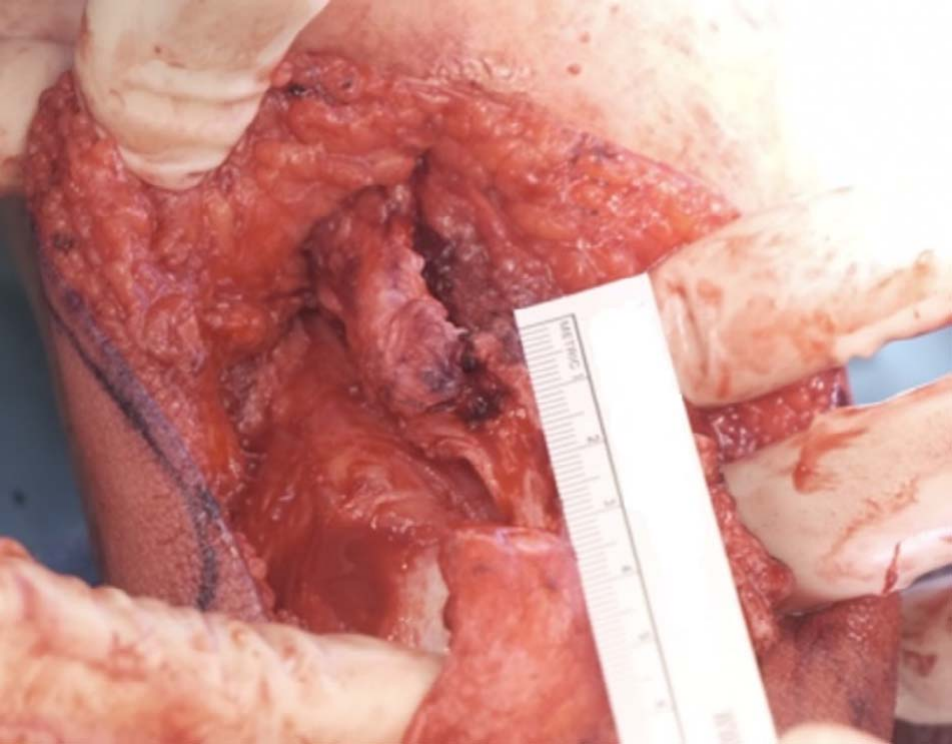
The measured Z-lengthening longitudinal cut is 3.5 cm.

The quadriceps tendon was exposed, and a clamp was placed under the tendon. The tendon was cut longitudinally, and 2 perpendicular cuts were made to this incision: laterally on the proximal end and medially on the distal end. This cut allowed a total lengthening of the quadriceps tendon of 3.5 cm (Figure 9).

**Figure 10.**
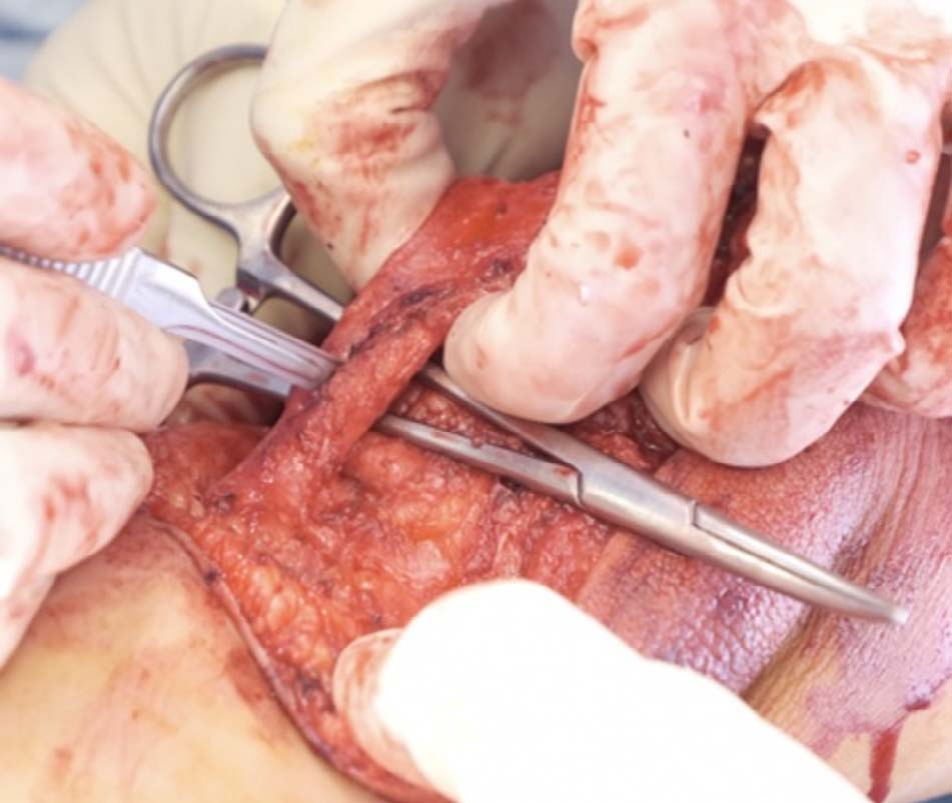
The patellar tendon is split and transected.

A split patellar tendon transfer of the medial side was performed with a clamp under the tendon for stability. The patellar tendon was transected, and the medial half was prepared for transfer (Figure 10).

**Figure 11.**
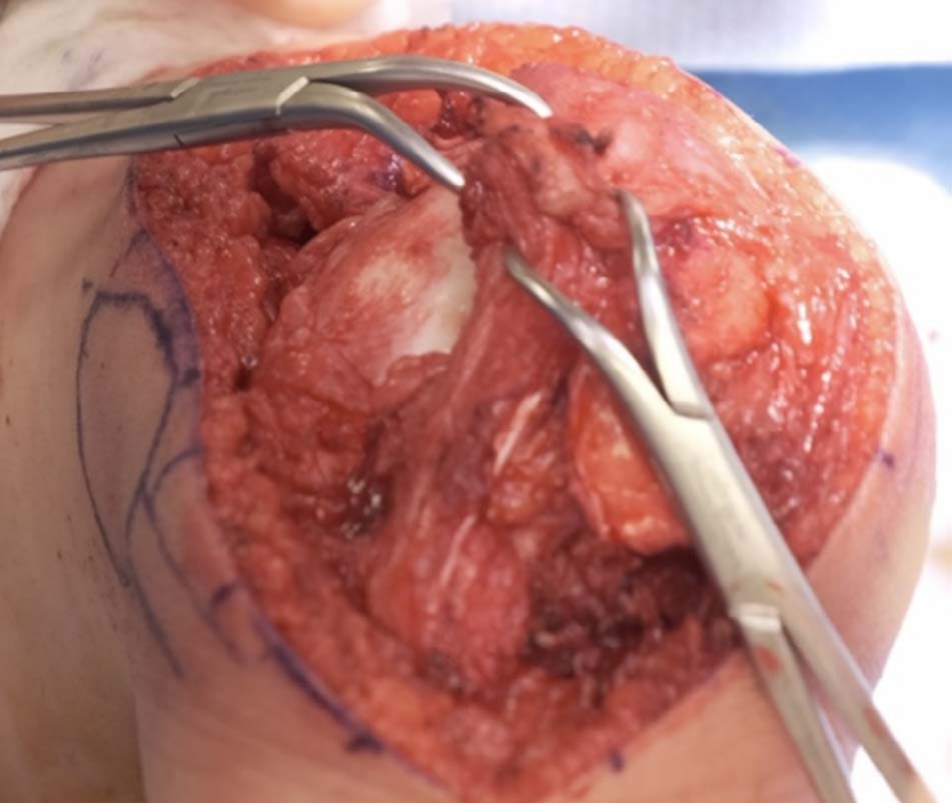
The vastus medialis oblique muscle advancement of 100% is held in place with clamps.

The patient's foot was placed on the sandbag with the knee flexed to 60° to allow reattachment of the tendons under proper tensioning. The vastus medialis oblique muscle at this time could be advanced with up to 100% overlap on the patella (Figure 11) and was clamped in position with the patella aligned over the trochlear groove. Correct tensioning of the vastus medialis oblique muscle was tested after clamping by watching the position of the patella in the trochlear groove as the knee was put through range of motion (ROM).

**Figure 12.**
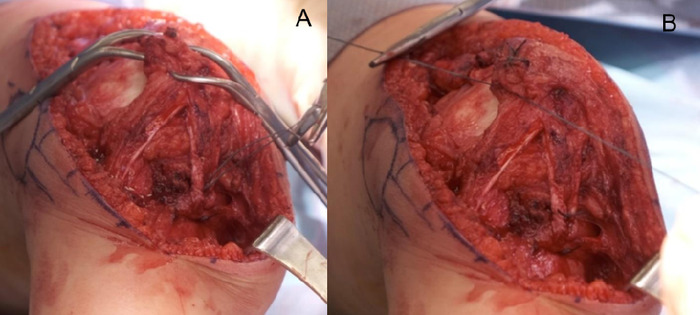
(A) The patellar tendon, now shaped like a V, is stitched to the pes anserinus. (B) The vastus medialis oblique muscle is mattress stitched to the patella.

The quadriceps tendon was reattached with the 3.5 cm gained from lengthening. The medial half of the patellar tendon was sutured to the pes anserinus. First, size 0 braided polyester suture was whipstitched into the tendon and then secured with proper tension (Figure 12A). The vastus medialis oblique muscle was then mattress stitched to the patella using size 0 braided polyester suture (Figure 12B). The patella was observed to sit well centered in the trochlear groove.

**Figure 13.**
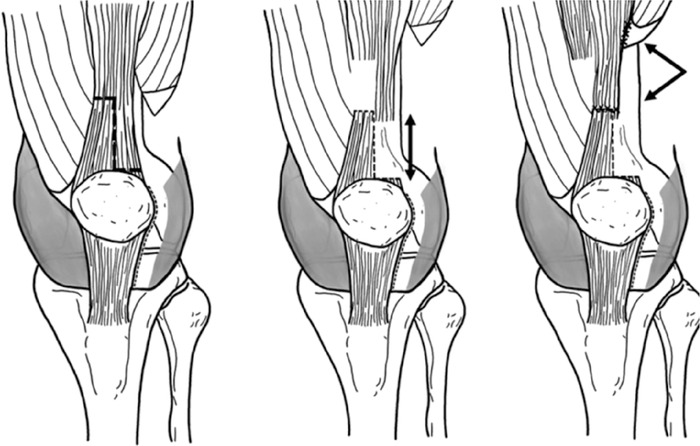
**These illustrations demonstrate the Z-lengthening procedure. Once the vastus lateralis muscle is released, the quadriceps tendon is cut along the dashed line, creating a Z. The distal piece of the Z remains attached to the superomedial aspect of the patella, with both pieces having a similar width. The 2 pieces are moved in opposite directions, with the arrow in the middle figure representing the quadriceps tendon length gained. The proximal and distal ends of the cut quadriceps tendon are whipstitched, and the vastus lateralis muscle is reattached to the lateral portion of the main quadriceps tendon. The arrows in the left figure indicate the location of both attachment sites.** (Adapted with permission from Ellsworth et al^10^ under Creative Commons license CC BY 4.0.)

**Figure 14.**
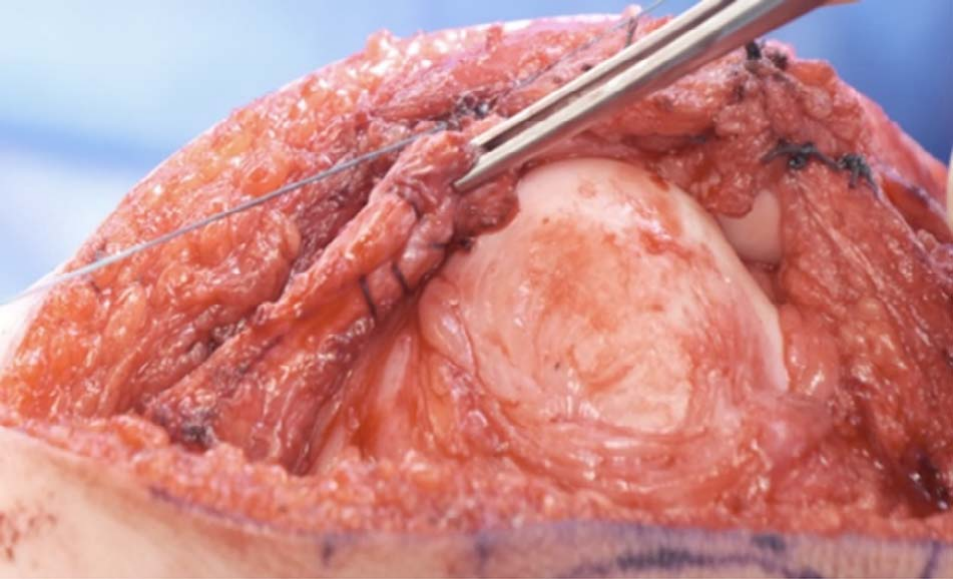
The proximal and distal ends of the Z-lengthened quadriceps muscle tendon are sewed together end to end.

The Z-lengthening of the quadriceps tendon was secured by whipstitching both the proximal and distal limbs of the quadriceps tendon with size 0 braided polyester suture. The proximal and distal limbs were sewed end to end (Figure 13^10^ and Figure 14).

**Figure 15.**
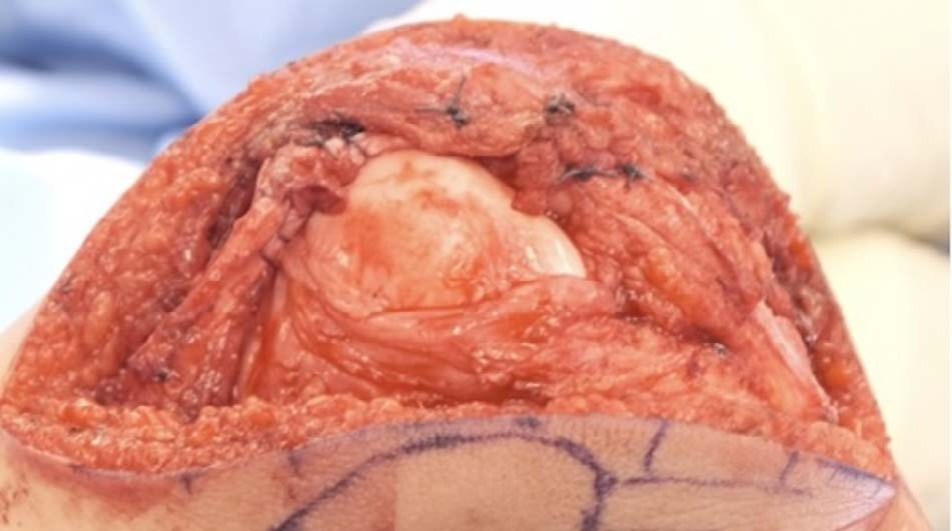
When the range of motion is tested, the patella stays centered in the trochlear groove.

The knee was put through ROM, and the patella stayed centered in the trochlear groove. The lateral aspect of the patella was located at the lateral border of the femoral condyle (Figure 15). Radiography confirmed this position.

### Postoperative Care and Follow-Up

Both patients had frequent follow-ups to assess ROM, weight-bearing, and scarring of the incision. ROM was assessed using passive motion, and weight-bearing was observed with and without crutches or immobilizers. Postoperatively, the ROM goal for both patients was 30° by 2 weeks, 60° by 4 weeks, and 90° by 6 weeks.

Both patients were instructed to remain non–weight-bearing with crutches and in a knee immobilizer until they were transitioned to weight-bearing. This instruction was not strictly followed, but lack of compliance was not concerning because the knee immobilizer allowed the patient to bypass the patella reconstruction when weight-bearing. Patient 1 was allowed to bear weight with the knee locked in extension at 2 weeks. Patient 1 used a continuous passive motion machine, but Patient 2 was too small to use the device. Both patients began physical therapy at 4 weeks postoperatively.

**Figure 16.**
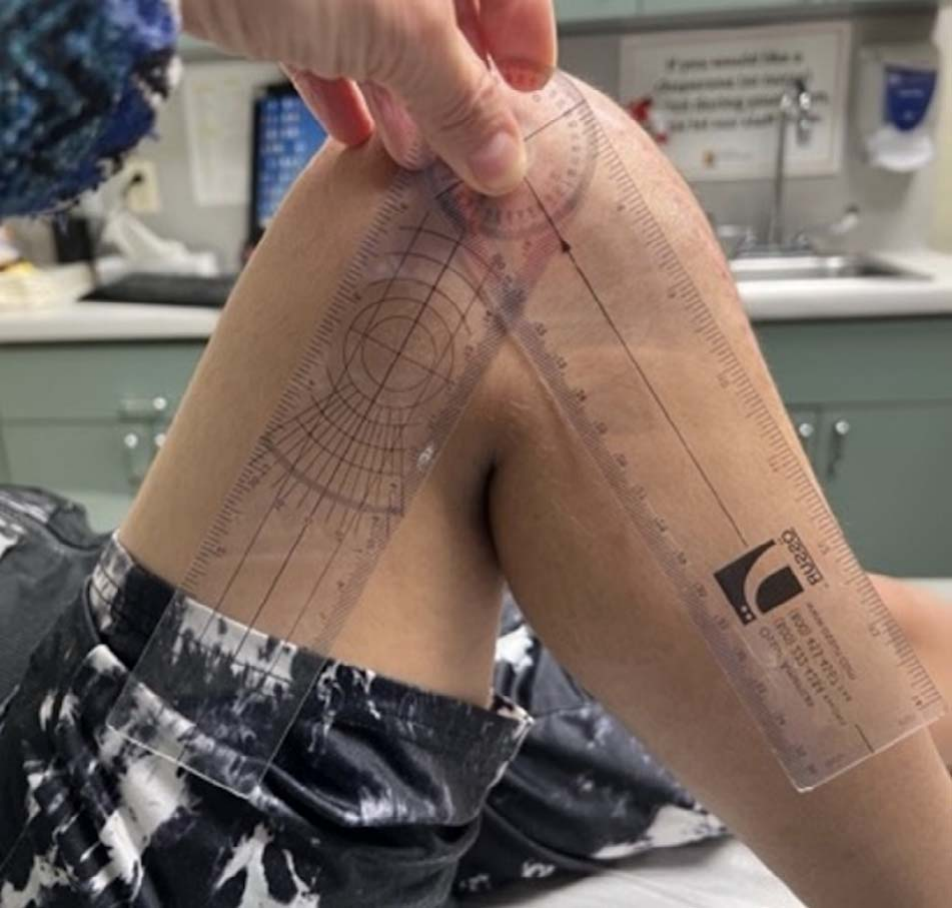
Eight weeks postoperatively, Patient 1 reaches 120° of flexion.

**Figure 17.**
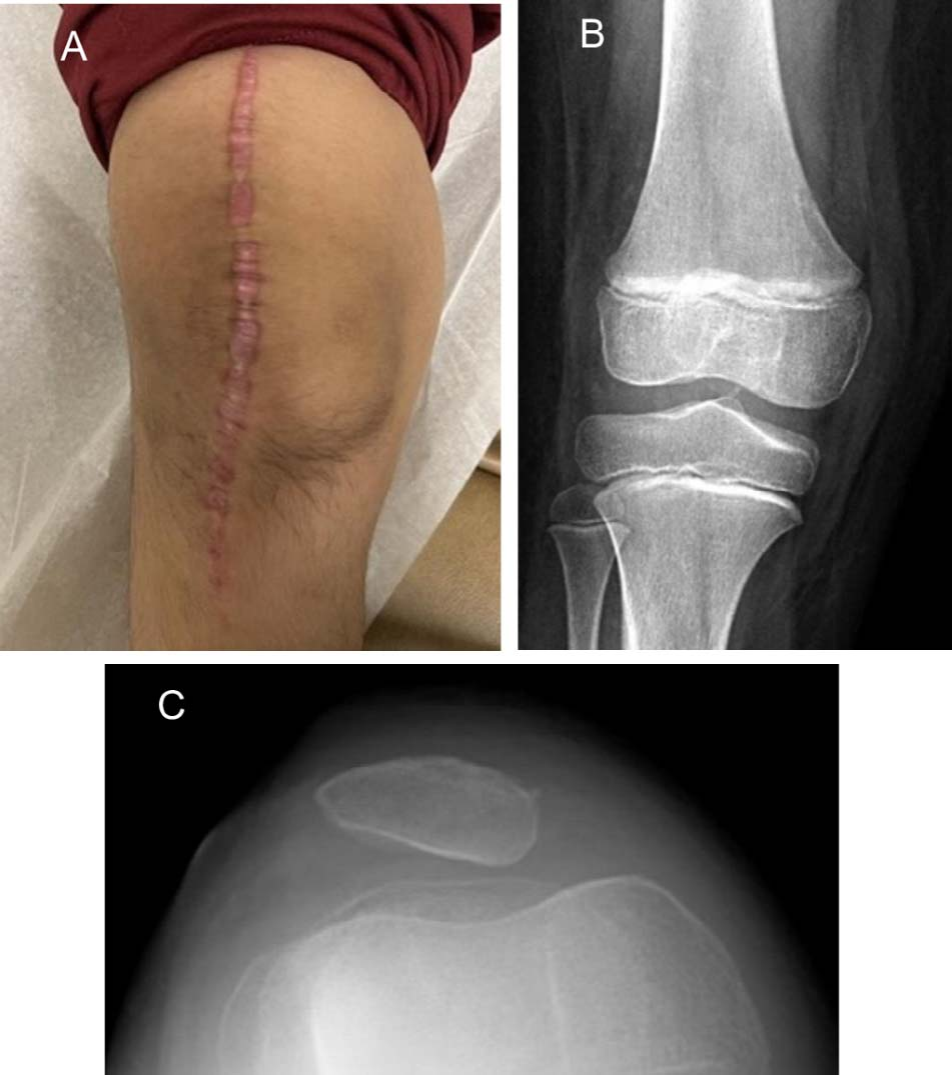
(A) Eleven weeks postoperatively, Patient 1 has no swelling, and the incision is healed. (B) Anteroposterior and (C) sunrise views of the knee at 11 weeks postoperatively show excellent alignment.

By 8 weeks postoperatively, Patient 1 met expectations, reaching 120° of flexion. He discontinued the knee immobilizer and acknowledged decreased pain and swelling, with some weakness during active flexion (Figure 16). At 11 weeks, Patient 1 attained full ROM with active and passive flexion to 165°. He had no weakness with active flexion and extension, and pain and swelling had completely resolved (Figure 17A). His patella was stable and midline, with x-rays showing excellent alignment (Figures 17B and 17C). A small click with motion was present when evaluating ROM.

At 6 months, Patient 1 had no activity restrictions, clicking of the patella, or complaints. He started playing sports for the first time in his life. At 1 year, Patient 1 continued to have no activity restrictions. He runs with no clicking, and the patella tracks medially. A summary of the treatment and follow-up timeline for Patient 1 is presented in Table 1. Patient 1 was satisfied with his fast recovery. Prior to surgery, he was unable to play soccer, but he is now fully active and thrilled to be able to participate in sports.

**Table 1.** Timeline of Treatment and Follow-Up, Patient 1

4 months preoperatively	The patient presents to the outpatient office with localized, intermittent right knee pain with an antalgic gait. The patella dislocates laterally with knee flexion. The patient cannot flex past 30° without full lateral patellar dislocation. He has no history of trauma to the knee.
3 months preoperatively	Magnetic resonance imaging shows the right patella dislocates laterally with an intact medial retinaculum. The treatment decision is surgery.
Day of surgery	The patient undergoes a procedure for soft tissue lateral release, Z-lengthening of the quadriceps tendon, medial transfer of the patellar tendon, and vastus medialis oblique muscle advancement.
2 weeks postoperatively	The patient is non–weight-bearing with crutches and a knee immobilizer. After his 2-week follow-up, the patient is allowed to weight-bear with the knee locked in extension. He uses a continuous passive motion machine.
4 weeks postoperatively	The patient is weight-bearing with a knee immobilizer. He is capable of flexion up to 70°. Physical therapy begins the day after this postoperative appointment.
8 weeks postoperatively	The patient meets the goal of 120° of knee flexion and discontinues the knee immobilizer.
11 weeks postoperatively	The patient has full range of motion with active and passive flexion to 165°. He has no weakness with active flexion and extension, but a small click is observed with range of motion.
6 months postoperatively	The patient has no restrictions with activity, clicking, or complaints. He is playing sports for the first time ever and is ecstatic.
1 year postoperatively	The patient continues to have no activity restrictions, clicking, or complaints. He continues to play sports and is fully active.

**Figure 18.**
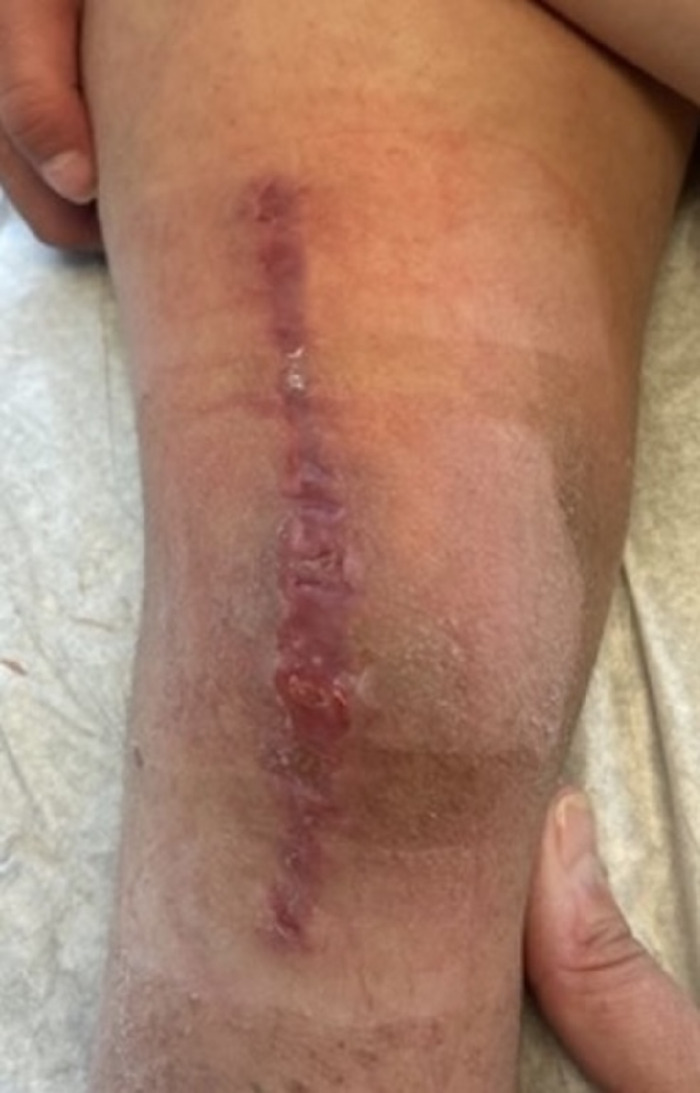
Six weeks postoperatively, Patient 2 has a closed wound with no signs of infection.

Patient 2 attained 90° of flexion by postoperative day 12; ROM progressed faster in this patient with Down syndrome because of hypermobility and reduced response to pain. However, Patient 2 had delayed wound healing because of noncompliance in protecting the knee. He flexed and kneeled on his knee, applying pressure to the incision. The patient needed constant supervision. The knee immobilizer was reapplied at a 3-week follow-up to protect and decrease tension on the healing incision. Prophylactic antibiotic coverage was initiated (cephalexin 25 mg/kg/d; 3.5 mL every 8 hours, 3 times daily) and continued for 2 months. Hydrocolloid dressings were used for wound care. At the 6-week follow-up, the wound was closed with no signs of infection (Figure 18). The scar was maturing at the 2-month follow-up. The patient began using kneepads when crawling. Knee immobilizer use was continued as the wound matured and was fully discontinued shortly before the 4-month follow-up.

At his 9-month postoperative follow-up, Patient 2 was still using a gait trainer for standing and bilateral ankle foot orthoses to keep him off his toes. He continued to work on his ability to walk with full knee flexion to 160°. A slight click was noted in the knee with ROM despite the stability of the patella because of muscle weakness after surgery. Patient 2 continued to use kneepads for crawling even after the scar was healed and mature.

At the 10-month postoperative follow-up, the patella remained stable, and the clicking originated from the femur on the tibia rather than the patella on the femur. The patient continued to use a gait trainer and ankle foot orthoses for ambulation. Patient 2 is now able to stand with stability. A summary of the treatment and follow-up timeline for Patient 2 is presented in Table 2.

**Table 2.** Timeline of Treatment and Follow-Up, Patient 2

11 months preoperatively	The patient presents to the outpatient office unable to ambulate. The parents observe a laterally dislocated patella and said the patient would “hit the side of his patella” to weight-bear in a standing frame. The patient has 15° flexion contracture. Surgery is discussed; the patient's family decides to obtain a second opinion.
3 months preoperatively	Magnetic resonance imaging shows the right patella dislocates laterally with an intact medial retinaculum. The treatment decision is surgery.
Day of surgery	The patient undergoes a procedure for soft tissue lateral release, Z-lengthening of the quadriceps tendon, medial transfer of the patellar tendon, and vastus medialis oblique muscle advancement.
12 days postoperatively	The patient attains 90° of flexion, but wound healing is delayed because of the patient's noncompliance by crawling on his knee.
3 weeks postoperatively	The patient is weight-bearing with a knee immobilizer reapplied to promote wound healing. The knee immobilizer was used intermittently to prevent crawling when the patient was unsupervised. Because of incomplete healing of a small area (<0.5 cm) distally and medially, a hydrocolloid dressing is applied to the incision and covered with cling wrap. The patient's parent applies the cling wrap to prevent the patient from pulling off the hydrocolloid dressing. The parents are instructed to change the dressing every 2 to 3 days. The patient is started on prophylactic cephalexin 25 mg/kg/d (3.5 mL every 8 hours, 3 times daily).
4 weeks postoperatively	Physical therapy is initiated to improve flexion and range of motion.
6 weeks postoperatively	The patient is weight-bearing with a knee immobilizer. No necrotic tissue is observed, and the wound is smaller at the medial part of the incision. The remainder of the incision is closed with no signs of infection. Cephalexin and hydrocolloid dressings are continued. The patient is cleared to take breaks from the knee immobilizer to work on motion and to prevent stiffness.
2 months postoperatively	The patient is minimally weight-bearing in a knee immobilizer with a standing frame. The incision is healed and entirety closed, and the scar is maturing with no signs of infection. Cephalexin is discontinued, but the hydrocolloid dressings are continued for 1 month. Knee immobilizer is transitioned to use only during sleep.
3 months postoperatively	The patient begins using kneepads when crawling to protect the incision.
4 months postoperatively	The knee immobilizer is discontinued shortly before this follow-up.
9 months postoperatively	The patient is using a gait trainer and ankle foot orthoses for ambulation. He has full flexion to 160°, and work continues on his ability to walk. A slight click is noted with range of motion.
10 months postoperatively	The patella remains stable. Kneepads are discontinued. The patient is still using a gait trainer and ankle foot orthoses, but he can stand with stability.

Patient 2 is nonverbal and unable to give his perspective, but his mother is happy that he can no longer voluntarily dislocate his patella.

## DISCUSSION

This case series illustrates the technique and advantages of the combined proximal and distal soft tissue realignment of the patella. During the procedure, the soft tissue was addressed in a systematic fashion, from the lateral to the medial side. The procedure is a variant of the Stanisavljevic procedure, which is a combination of the Roux-Goldthwait technique and a knee joint capsule advancement. Stanisavljevic et al used a release of the vastus lateralis muscle, a patellar tendon transfer of the lateral half pulled under the medial half, and a complete coverage of the patella with a thick tissue capsule.^3^ The Roux-Goldthwait technique employed a longitudinal dissection of the patellar tendon, removing the lateral half of the patellar tendon from the tibial tuberosity to shift this portion of the patellar tendon medially under the medial half of the remaining nondetached tendon. This lateral piece of the tendon was then sutured to the pes anserinus on the medial side of the knee.^11^ Historically, the Roux-Goldthwait technique has been shown to result in patellar tilt by pulling the lateral half of the patellar tendon under the still-attached medial half, resulting in torque.^1,4^ The joint capsule advancement enables tightening of the medial capsule by shifting it into anatomic position.^3^ Fixing the patella inside the capsule is a crucial component to the success of the Stanisavljevic procedure to provide sufficient medial support and stability. Furthermore, use of the joint capsule has been changed to dissection of the vastus medialis oblique muscle off the patella, and the vastus medialis oblique muscle is then brought over the patella by 50% to 100% coverage. The vastus medialis oblique muscle is sutured in place, holding the patella in the anatomically correct position. In this case series, vastus medialis oblique muscle advancement facilitated optimal patellar tracking and sustained postoperative stability of the knee for both patients.

Multiple adaptations have been made to address the complications of the Stanisavljevic procedure, and the approach described in this case series represents an amalgamation of techniques aimed at optimizing patient outcomes. Our surgical approach integrates multiple proven operative techniques into a single procedure, offering advantages over previous methods. Prior studies have demonstrated that ODP often requires a combination of surgical strategies because of the severity of the condition.^2,5^

One advantage of our surgical technique is achieving patellar stability through transferring the medial half of the patellar tendon rather than the lateral half. The average follow-up in the Gordon and Schoenecker study of patients surgically treated for congenital dislocation of the patella was 5.1 years, with a range of 1 to 17.5 years.^1^ By their final follow-up, all patients reported reduced pain and enhanced activity tolerance. However, Gordon and Schoenecker noted in their early cases that a Roux-Goldthwait repair of the patellar tendon using the lateral half of the patellar tendon did not work well, and they subsequently revised their surgical technique to a medial transfer of the patellar tendon.^1^ We used this improved technique for our patients, and the result was normal patellar alignment without tilt. Maintaining the lateral half of the patellar tendon helps preserve tension in the knee when reconnecting detached structures, such as the medial half of the patellar tendon, vastus medialis oblique muscle, and quadriceps tendon. Preserving the integrity of the patellar tendon enables initiation of early weight-bearing and flexion, as seen in our patients.

Gao et al reported using the Stanisavljevic procedure in 12 patients with congenital dislocation of the patella, with the addition of lengthening the rectus femoris muscle to address tightness in the extensor mechanism.^2^ The surgery was successful in 10 patients; the other 2 patients had unsuccessful procedures, theorized to have been caused by inadequate release of the tight extensor mechanisms. Our technique addresses the contracture of the quadriceps muscle through Z-lengthening the quadriceps tendon, increasing the tendon length to optimize functional strength with movement.

Wada et al described a technique similar to the Stanisavljevic procedure, adding a V-Y lengthening of the quadriceps tendon in patients presenting with quadriceps muscle contracture (increased stiffening of the quadriceps muscle).^5^ Wada et al only used V-Y lengthening in patients with knee flexion contracture of ≥10°, a decision that limited the benefits for patients with low to mild knee flexion contracture.^5^ In the Wada et al study, most patients used bracing before their procedure which stabilized improvement of their flexion contracture.^5^ Both of our patients, regardless of severity of knee flexion contracture, were treated with a Z-lengthening of the quadriceps tendon, improving tendon length and flexion. Patient 1, who presented with no knee flexion contracture, now plays sports without the risk of developing a tight quadriceps muscle. In Patient 2, who presented with knee flexion contracture of 15°, knee flexion has improved. In both patients, Z-lengthening of the quadriceps tendon increased tendon length, helping to achieve a more normal knee anatomy. A generous lateral release of the retinaculum and a release into the lateral quadriceps muscle are required for operative repair. Extending the dissection into the lateral quadriceps muscle fully relieves lateral tightness when contracture is encountered. Following this extensive lateral release, both patients exhibited stable patellar alignment and no recurrent dislocations, supporting the clinical benefit of an extensive lateral release in relieving lateral constraint.

Possible complications with this surgical procedure include continued patellar dislocation, perioperative bleeding, stiffness of the knee postoperatively,^2^ infection of the wound,^12^ and chronic knee pain.^4^ In Patient 2, contraction of his lateral quadriceps muscle still causes a minimal amount of lateral pull on the patella. However, because the lateral retinaculum was released into the quadriceps muscle during the surgical procedure, the patient can no longer dislocate his patella. A small click is still present but may disappear over time with further strengthening of the ligamentous and muscular support of the knee.

As our patients reach maturity, they may experience patellar pain or subluxation. They may need to undergo bony procedures to address issues such as valgus deformity or tibia rotation. Additionally, our patients may need reconstruction of the medial patellofemoral ligament for added medial support.

## CONCLUSION

The surgical technique described in this case series—incorporating extensive lateral release, quadriceps tendon Z-lengthening, patellar tendon split/medial patellar tendon transfer, and 100% vastus medialis oblique muscle advancement—addresses the limitations of previous procedures and supports long-term positive outcomes for both of our patients with ODP. By correcting both proximal and distal soft tissue abnormalities, this combined approach effectively manages the complex pathology associated with lateral patellar dislocation and mitigates the risks of patellar tilt, knee flexion contracture, and recurrent instability. We recommend considering this soft tissue technique for all skeletally immature patients to stabilize the knee and reduce the likelihood of reoperation. The outcomes of our procedure—enhanced activity tolerance, expedited ROM recovery, and improved joint stability—underscore its value as a practical and reproducible option for complex patellofemoral pathology.

## Notes

*This article meets the Accreditation Council for Graduate Medical Education and the American Board of Medical Specialties Maintenance of Certification competencies for Patient Care and Medical Knowledge.*
